# Expulsion of Mr. Lambert, with Reflections and Observations

**Published:** 1829-04-01

**Authors:** 


### XVI.
### MEDICAL SOCIETIES, LONDON AND WESTMINSTER.

"It is still an odious office; but I know not who ever succeeded in steering exactly between that criticism which the apposed party brands with the term acrimony or worse, and that timidity or over-caution which defeats the object in view; leaving truth to quail and fly before the enemy to which it shows a misplaced tenderness."—*Macculloch.*

MEDICAL degradation has passed its zenith, and is fast vanishing into the regions of darkness whence it sprang—the star of medical honour is on the ascendant. The late trial has had a most beneficial effect in rousing the minds of medical men to a sense of the degeneracy into which they were plunging themselves by tolerating, and thereby encouraging the system of personal defamation which had usurped the name and character of legitimate criticism and wholesome censorship. The press ran riot, in consequence, and at last so completely outraged public feeling, that strong reaction ensued. The verdict of the jury, but still more the disclosures made in court, opened the eyes of the profession, and the question generally asked by every one, "how are we to shew our detestation of the libel?" was naturally answered, "*by avoiding the society of the libeller.*" This might be readily done in private life. But there was a higher duty to perform. The Westminster Medical Society led the way in discharging this duty, and in such discharge they have done themselves immortal honour, and conferred a lasting obligation on the profession.

What! are we to have no laws but the statute laws in medical society? Is every man, who can keep his neck out of a halter, or his feet out of the stocks, to be considered as perfectly fit for association with professional men? If the president of the Westminster or London Medical Society took it into his predilection to steal sheep, and served an apprenticeship of 14 years in Australia for that predilection, would he claim the chair of either society on his return—on the plea that he never purloined a single wether or mutton, of any kind, from Sackville Street or Bolt Court? The thing is preposterous. It outrages common sense, and the two societies have shewn their appreciation of the argument. The constant plea set up by Mr. Lambert and his advocates is this—"Have I committed any act against the

Westminster or the London Medical Society?"—"What specific charge can be brought against me?" We answer in a single line from the Roman poet—

"Quæ regio in terris '*vestri*' non plena '*pudoris*.'"

We accuse him, not of any crime cognizable by the law—not even of LIBEL, because men of the most honourable character have been guilty of that*—but we accuse him of acting in the most *unprofessional* manner—of thereby lowering the medical character in the eyes of the world—and consequently of outraging every feeling of honour in his professional brethren. The simple question is this—has Mr. Lambert degraded himself, or been degraded in the estimation of his fellow practitioners, and of society at large, by certain recent transactions? We ask every man capable of reflection this question, and we abide by the decision.

Is it no degradation to be turned out of three public hospitals? Is there an instance on record where a medical student or a medical practitioner was ever so served—for good conduct? Is it no degradation to be told by a judge on the bench, that he never answered a question straight forward like a man?† What did Lord Tenterden mean by this? The advocates of Lambert will say that he answered like—an ANGEL! We want no such angels in our medical societies.

Was the testimony of Dr. Hodgkin and Mr. Key no degradation to Mr. Lambert? Did they perjure themselves when they swore that there was no hole between the bladder and rectum before Lambert handled the parts? What says the *straight-forward* man to this? He states that his finger went through the cellular tissue without the slightest impulse from his hand. No force—oh no, on the honour of a Lambert, there was not an atom of propulsion given to the finger. It went up of its own accord, and not only so, but dragged the hand and arm behind it! There is a new law in animal mechanics for the next edition of Dr. Arnott's book! But the advocates of Lambert will assert that, the FINGER, on this occasion, was laudably employed in making anatomical discoveries. Yes, it did make a discovery! Well may Mr. Wakley say that—"Mr. Lambert's name will stand conspicuous in the surgical annals of his country," when the members of the Westminster Society will be "rotten and forgotten." No doubt of it. This discovery alone will immortalize him beyond a Harvey and a Jenner! How shall we announce it. The FORAMEN LACERUM in BASI—CADAVERIS!! There's a discovery for the 19th century! How will the foramen Monroianum shrink into insignificance, when compared with the "FORAMEN LACERUM LAMBERTI!*"

But to come to a tangible charge. Has not Mr. Lambert been degraded beyond all measure by the REPORT itself, leaving every other consideration out of the question? Why, the potatoe-merchant (his own witness) was ashamed of it, and pronounced it "*a most unprofessional report.*" Is there a stone in the streets of London—is there a foot of ground between Camberwell and Highgate, that would not cry shame (if they had tongues) when trodden on by the REPORTER of Mr. Cooper's operation! Even if the report were as true as it is false, the stigma incurred by its construction could never be obliterated. And was the WESTMINSTER MEDICAL SOCIETY to continue this reporter on their rolls—to sit and converse with him—to associate with him, and thus sanction his

* Mr. Lambert thought he had hit on an excellent plea of defence, when he asked why Dr. Johnson was not turned out of society, because he was convicted of a libel! What a damning satire did he here pass on himself! Yes, let the question be asked from Cancer to Capricorn—from the Orontes to the Ohio—and the answer will be very short:—the circumstances were not *precisely* similar—neither are the individuals.

† By some strange mistake in the short-hand writer, (it was not Mr. Gurney who took down that part of the evidence,) the rebuke of Lord Tenterden is quite altered in Mr. Cooper's edition of the trial. But we heard the words, and can swear to them. We appeal to every man in Court for the truth of this. In the Westminster Society Mr. Lambert did not attempt to deny it, but excused himself thus:—"Suppose that if I had gone into the witness-box and sworn *straight forward*, through thick and thin, without any deliberation," &c.—*Lancet*, *Jan.* 10, 1829. But, allowing that the version given in Mr. Cooper's edition is correct, (which we deny,) Mr. Lambert has gained very little. The judge told him that, instead of answering directly, he kept "*fencing*" about, which was "most unbecoming." Utrum horum mavis accipe!

* His friend Dewhurst will correct our nomenclature, if we are in error.

conduct to a brother practitioner? No. The whole influence of the LANCET was exerted on a society which contains nearly a thousand members, and all the Lancet could muster was *nineteen* votes out of NINETY-FIVE members who advanced to the poll! Yet this astounding expression of public feeling did not open the eyes of either Wakley or Lambert. They both appealed "to the surgeons and *general practitioners*" (as if the latter were not alive to the degradation attempted to be put upon them) to come forward and oppose the confirmation of the minutes. What was the result of this second appeal? Mr. Lambert was turned out of the society, amid the hootings and hissings of an hundred tongues! When the members were called on for their confirmation of the expulsion, every hand was up—when the negative was put from the chair, not a human finger was raised in support of the SUB-EDITOR of the LANCET!!!

This unhappy man appears to be lost to all sense of shame. In the speech which he made in his defence, he asserted, (by way of shewing that he bore no malice to Mr. Cooper,) that he had corrected Mr. Cooper's work on (we believe) the ligaments! Now this must have been a deliberate invention, poured forth to answer the object of the moment, without the slightest regard to the detection which was instantly to follow. Mr. Cooper immediately shewed the mendacity of the statement in the succeeding MEDICAL GAZETTE, and branded him with the falsehood in the London Medical Society! Ex his disce omnes.

But we shall add another trait to the portrait. Every body knows what credit this Lambert takes for always acknowledging himself the reporter of Mr. Cooper's operation, and of the Borough hospitals generally. On the day the report was published he went up to Mr. S. and the surgery-man of Guy's Hospital, who were talking together, and asked them if they had seen the Lancet? On being answered in the negative, Lambert observed that there was in it a heavy charge against Bransby Cooper, and as he was suspected of reporting for the Lancet, the narrative of the operation would probably be laid at his door. Now, says Lambert, I CALL HEAVEN TO WITNESS THAT I NEVER WROTE A LINE OF THE REPORT, and I request you will contradict the suspicion in every direction!!*

* This was offered to be substantiated by two affidavits in the London Medical Society.

Such is the sub-editor of the Lancet—the sub-reformer of the surgical profession—the *self-appointed* second in command, the lieutenant-colonel of a corps which he has been enabled to insult, but failed to disgrace—the GENERAL PRACTITIONERS OF ENGLAND! No man is bound to acknowledge himself the author of an anonymous publication—nay, he may deny the fact, if impertinently questioned on the subject. But any man who writes an anonymous paper, and then goes about *voluntarily* protesting to Heaven that he is not the author, commits as great a breach of honour, and, we believe, of morality, as if he took a false oath in a public court.

Next let the reader turn to Lambert's speech, as reported in the Lancet, and he will find this "straight forward" man appealing to the "great body of the society, consisting, as he believed, of *independent and impartial men*." When they decided on his expulsion, and he was conducted out of the room by police-officers, this same man cried out, while running down stairs—"you are a pack of bl——ds!!!" Such is the *gentleman* who sub-conducts the Lancet, and who is lauded by his gentlemanly master for an "*undeviating course of honorable conduct*."!! The public must entertain profound respect for the assertions of that man who can, at one moment, characterise a medical society as consisting of "independent and impartial men"'—and in the next breath as—"a pack of bl——ds;"—who can *deliberately* indite such a report as that of Mr. Cooper's operation, and then *voluntarily* appeal to the throne of his Creator as witness, that he never wrote a line of it! Three fourths, at least, of the Westminster Medical Society, are highly respectable GENERAL PRACTITIONERS. To these Lambert appealed through the medium of the Lancet, as well as in his speech. They were independent and impartial before they gave their votes—they were bl——ds immediately afterwards!!

But there may be men who conceive that the medical profession is standing rather too high in the estimation of society at large—that we are raised quite above our business—that we have got into the parlour, when we ought to be in the pantry—that we associate with men of science, literature, and philosophy, when we ought to be associating with their butlers, cooks, and footmen. They conceive that we have no occasion for barons and baronets in the medical profession. We want not your Sir Henry Halfords, your Sir Astley Coopers, your Sir Gilbert

Blanes, or your Sir James M'Grigors—your Baron Larreys, your Baron Boyers, or your Baron Dupuytrens. Oh no!—the "average maximum" of medical respectability is to be sought among your Lamberts, Lees, Claphams, Thomas's, Gilberts, and Wakleys! Men of "warm feelings," and fertile imaginations; who can *dramatise* a case of lithotomy, and work up a *finger-scene* equal to the handkerchief of Othello:—Public functionaries; that is to say, PUBLIC EXECUTIONERS, who can earn eight guineas a month by PHLEBOTOMY alone—by bleeding their neighbours! besides extra-wages for all capital operations, such as trepanning, amputating, castrating, and—CALUMNIATING! Yes, we must look for the "average maximum" among men whose genius can unite the lucrative avocations of PILL and POTATOE merchant—among youths who are so eager to enter on *practice* under the age of twenty, that they begin by—swallowing a *black dose* before a magistrate, without making a wry face—among such eminent personages as TIN-MEN'S apprentices, who are determined to make a noise in the world in some way or other—among your "hard-featured" hostlers of country inns, who, assisted by Mr. Wakley, can tie the subclavian artery "by accident!"—Yes, the medical profession will never be on a proper footing, till it is brought up to the level of the above-mentioned standards of perfection. We conjure the "SURGICAL REFORMERS," therefore, to flock round the ensigns of Messrs. Wakley, Lambert, and Haslam, as leaders, who must insure victory, wherever they unfurl their flags. The members of the WESTMINSTER MEDICAL SOCIETY being a pack of "independent and impartial" "bl——ds," are to be expelled from the community of physic, and annihilated forthwith, by Mr. Wakley's determination never more to publish their proceedings!

We now come to the London Medical Society, the most ancient, and, till Haslam presided over it, one of the most flourishing medical societies in Europe. How the quondam Doctor of Bedlam got into the chair, we know not; but we give ourselves some credit for divination, when, as is well known, we long ago demurred to the station which Haslam occupied.* Our readers are aware that we conjured the council to bestir themselves, and resent the oppressive conduct of their chairman. They have done so; and Haslam, after supporting the sinking fortunes of Lambert as long as possible, by every kind of sophistry and bad manners, has prudently resigned. His final words are remarkable. "Of *moral* courage, I possess sufficient for all the honorable purposes of civilized society; but I freely confess that I am a stranger to the cold-blooded diplomacy, that enables a human being to become the presiding minister of injustice, and I want nerves to witness the character of an innocent person, mangled and lacerated by his own brethren, as an expiatory sacrifice for a disastrous operation."

We were obliged to read the above passage twice over, before we were satisfied that it was not meant to convey a cutting satire on Mr. Lambert. Whose character has been mangled and lacerated by his brethren, on account of a disastrous operation? Mr. Bransby Cooper's! Who was the mangler? Mr. Lambert! Yet this worthy President (that was) pretends to shed crocodile tears of sympathy for the "MANGLER," because he is called to account for writing the most diabolical report that was ever scrawled in the ink of defamation!! This President Haslam, after endeavouring to brow-beat the Council of the London Medical Society, consisting of most honorable and enlightened practitioners, had the INSOLENCE to read to the said Society, on the night before his resignation, the following prophetic *sentence* on his own presidential *life*. "Mr. WAKLEY introduced by MYSELF." What! did he suppose that the presence of this man would strengthen his nerves to support the "groans and hisses" of an insulted Society? If he did, (and there is no doubt that he did) he has been most miserably disappointed.

On Monday night, the 19th January, the society met, and was crowded to excess. The venerable Dr. Babington moved the expulsion of Mr. Lambert, in a speech as forcible as it was mild. Let the medical profession read this and engrave it in their memories! Dr. Babington, the patriarch

* The Editor of this Journal declared to Mr. Field (the Registrar) his determination to withdraw from the Society, so long as Haslam presided over it, more than twelve months ago:—though the London Medical Society was, and is, one for which he entertains the highest respect. But he would not consent to take part in a society over which a radical president held sway.

of Medicine, against whom the breath of scandal or the bias of party has never even insinuated a blemish, MOVED THE EXPULSION OF LAMBERT!! Will the rankest radical, after this, dare to say that Lambert's expulsion was a party question —an assertion made by a member of the Westminster Medical Society? A party-question! Between what parties? But, on reflection, we beg Mr. Dewhurst's pardon. It *was* a party-question! It was a party-question between—the good and evil principles of human nature—between man civilized and man savage—between truth and falsehood—between justice and injustice—between honour and perfidy! Yes! the LONDON MEDICAL SOCIETY has not merely rivalled but outstripped the Westminster, in marking its detestation of Mr. Lambert's conduct, and consequently of the principles of the Lancet. Of SIXTY-SEVEN members who went to the poll, only NINE voted against the expulsion, and most of these on account of some technical informalities in the summoning of the special general meeting. Thus Lambert was expelled from the Westminster Society by 4 to 1—from the London Society by 6 to 1. We regret that our limits prevent us from giving a full account of this meeting, but we cannot avoid adverting to one or two points. When the specific charge (and be it remembered that Lambert, Wakley, and Haslam were always calling out for *specific* charges) was made respecting Lambert's *voluntary* appeal to the throne of his Creator, that he never wrote a line of the REPORT, the "FENCER" (as Lord Tenterden called him) immediately objected to a *specific charge*, as none was stated in the indictment! A postponement of the investigation was then offered him, provided he pledged himself to meet the accusation of the *voluntary oath*, that he did not write the report. He was silent! He dared not deny the oath! Yet this is the man extolled to the skies by Wakley, as remarkable for his inflexible integrity and undeviating veracity!

We have now lived to see the day when the tide of evil passions has clearly begun to ebb, and when the tarnished honour of the Medical Profession in this country, is likely to recover its lustre. Englishmen have always been remarkable for good sense and sound judgment in the end, however prone they may be to ebullitions of feeling at the moment. Unless the national character be entirely altered—and unless the medical profession be inferior in understanding to the other classes of society, the age of defamation is hastening to a close, and the days of calumniators are numbered. Look at the LONDON MEDICAL SOCIETY. Who would not have supposed, from the reports (falsified or modulated no doubt) of the last year or two, that this society contained a considerable number of LAMBERTINES, with the President at their head, some of them standing candidates for a niche in the temple of fame on Saturday mornings? How changed is the scene! The moment that the respectable portion (and we are happy to say that 99 in the hundred of the Society are such) had their sense of justice and propriety roused, they manfully came forward, and not only detruded from their presence the CALUMNIATOR of one of their members, but openly and fearlessly expressed their abhorrence and detestation of the principles and practice of the Lancet. We have no hesitation in saying, that the LONDON MEDICAL SOCIETY has acted not only in strict accordance with professional honour, decorum, and etiquette—but with the utmost policy, in regard to its own welfare and existence. Men will now be proposed there, and will join the society, who are capable of doing honour to its meetings; but who would never have entered its walls under the Haslam administration. The late proceedings, too, have prevented, to our knowledge, some most worthy members from seceding. The spirit and good feeling evinced by the London and Westminster Societies, will operate like an electric shock through the whole of the profession. While no movement was made, no demonstration of abhorrence evinced in the metropolis, the provincial practitioners remained in doubt —and, at all events, thought it useless to commence a resistance at the periphery, when the centre was paralyzed. The decisive triumph of good over bad principles in the metropolis—the utter weakness, nay imbecility of the RADICALS, when brought into collision with the respectable classes of the profession, must silence the defamatory partisans in every province of the kingdom, and unite the advocates of order, decorum, and liberality, wherever the news extends.

We must now come to a conclusion; and we shall not easily be drawn into such lengthened articles on medical politics again. But we ask our professional brethren, how we could remain silent when events of such national importance were passing before our eyes? Shall we be censured for exerting our powers, and the powers of the press, in putting down the horrible system of moral assassination and professional murder, which has disgraced our age and country, when the venerable BABINGTON left a sick bed to raise his voice against the said system? To those well-intentioned friends who think we ought not to sully our pages with the mention of the Lancet and its advocates, the Wakleys, Lamberts, and Haslams of the day, we recommend a perusal of the epigraph which we have affixed to this article. We believe, however, that there is now but little need of our assistance. The professional spirit and good sense of Englishmen, when once roused, require no fuel to support the flame, and we shall now probably take leave of the Lancet for ever!

A review of our whole conduct, during a five years' contest with the organ of professional defamation, harrows not up a single unpleasant reflection. We denounced it in its origin—we opposed it in its progress—we defied it in its zenith—but we shall spare it in its fall.

---

